# Deciphering the Impact of High-Fat Diet and Tobacco Exposure on Urothelial Integrity and Bladder Inflammation: A Mechanistic Systematic Review

**DOI:** 10.7759/cureus.105057

**Published:** 2026-03-11

**Authors:** Hope O Francis, Worship O Agbonifo, Joseph Chimezie, Mercy Awoleye, Temitope Adedeji

**Affiliations:** 1 Physiology, Federal University of Technology Akure, Akure, NGA

**Keywords:** bladder inflammation, high-fat diet, lower urinary tract symptoms (luts), metabolic syndrome (mets), smoking tobacco, urothelial

## Abstract

Lower urinary tract symptoms (LUTS) are a major global health burden and are closely linked to urothelial dysfunction and chronic inflammation. Modifiable lifestyle factors, especially exposure to high-fat diets (HFDs) and tobacco products, appear to contribute to bladder pathology through overlapping biological pathways, but how they interact mechanistically is still not well understood. This Preferred Reporting Items for Systematic Reviews and Meta-Analyses (PRISMA) 2020-guided systematic review synthesized mechanistic and epidemiological evidence on how HFD and tobacco products affect urothelial integrity, bladder inflammation, and the development or progression of LUTS. PubMed/MEDLINE, Cochrane Library, Scopus, Web of Science, and Google Scholar were searched for 2012-2025 studies; 14 human studies and 18 animal model studies met eligibility, and findings were qualitatively summarized with risk-of-bias assessment using appropriate tools. Evidence consistently shows independent harms from both exposures, with HFD driving metabolic inflammation, oxidative stress, and hypoxia, causing urothelial barrier disruption, detrusor fibrosis, altered contractility, and increased cystitis susceptibility. Tobacco, including tobacco-specific nitrosamines (TSNAs) and nicotine, causes urothelial injury, DNA damage, chronic inflammation, and epithelial hyperplasia, aligning with worse LUTS and higher bladder cancer risk. Both act via shared mechanisms such as oxidative stress, nuclear factor kappa B (NF-κB) activation, and endothelial dysfunction, supporting the critical role of lifestyle interventions for bladder health.

## Introduction and background

Urothelial dysfunction and chronic inflammation within the bladder epithelium are central to the development of lower urinary tract dysfunction (LUTD), a spectrum of storage, voiding, and post-voiding disturbances resulting from impairment of the bladder, urethra, or prostate [1]. The urothelium plays an active role as both a barrier and a signaling surface [2]. When this barrier is compromised, increased permeability can trigger local immune responses and oxidative stress, which in turn contribute to changes in the structure of the bladder detrusor muscle [3]. Clinically, these bladder changes commonly present as lower urinary tract symptoms (LUTS), urinary frequency, urgency, nocturia, hesitancy, weak urinary stream, and incontinence, which substantially reduce quality of life and contribute to morbidity [4,5]. Growing evidence further links this multifactorial disorder to systemic metabolic dysfunction, chronic inflammation, and vascular impairment in men and women [6-8].

Lower urinary tract symptoms (LUTS) constitute a significant global health burden and represent the most common clinical entry point into LUTD evaluation [9]. Population-based evidence suggests that more than half of adults report at least one LUTS, with marked regional differences, highest in Africa (43.9%) and Europe (43.3%), and lowest in Oceania (20.3%) [10]. These symptoms increase steadily with advancing age and are more prevalent among men, largely due to prostate-related pathophysiology, although storage symptoms predominate among women.

Across major cohorts in high-income settings, LUTS affect both men and women, with nocturia and urinary frequency frequently reported and overactive bladder (OAB)-type symptoms present in a substantial proportion of adults aged ≥40 years [11,12]. Importantly, epidemiological patterns consistently associate LUTS with modifiable cardiometabolic and lifestyle exposures, particularly obesity and smoking, alongside hypertension and aging [13].

While LUTS capture the patient’s symptom burden, they do not, on their own, reveal the underlying cause [14]. Symptom-based scoring tools correlate imperfectly with objective urodynamic findings such as detrusor overactivity, bladder compliance, or residual urine volume [15]. This symptom mechanism gap makes it essential to frame LUTS within the underlying tissue-level drivers of LUTD, including urothelial barrier disruption, chronic mucosal inflammation, oxidative stress, neurohumoral dysregulation, and progressive detrusor and vascular remodeling [16].

Understanding LUTD and bladder dysfunction, therefore, increasingly requires a holistic approach that accounts for cumulative and interacting exposures across the lifespan [17]. Environmental, dietary, and behavioral stressors act synergistically with genetic and metabolic factors to influence bladder physiology [17]. Among these, high-fat dietary intake and tobacco exposure stand out as pervasive modifiable risk factors, each exerting systemic and local effects through overlapping biological pathways involving metabolic inflammation, oxidative stress, and vascular impairment.

High-fat diets (HFDs) promote metabolic dysregulation characterized by obesity, insulin resistance, and endothelial dysfunction, all of which can compromise bladder function. Experimental studies have shown that HFD feeding alters the expression of urothelial tight junction proteins (ZO-1 and occludin), increases macrophage infiltration, and heightens oxidative stress within the bladder wall [18]. Chronic metabolic inflammation induced by HFDs elevates systemic cytokines such as interleukin-6 (IL-6) and tumor necrosis factor-alpha (TNF-α), disrupts nitric oxide signaling, and contributes to detrusor fibrosis and ischemic remodeling [19]. Clinically, obesity, often a downstream effect of sustained high-fat consumption, has been linked to increased intravesical pressure, detrusor overactivity, and overactive bladder (OAB) symptoms, thereby exacerbating LUTS [20].

Tobacco use, another pervasive environmental exposure, exerts profound local and systemic effects on the bladder and urothelium [21]. Tobacco products can be broadly categorized into combusted forms (cigarettes, cigars, pipes, and waterpipe/shisha) and smokeless forms (chewing tobacco, snuff, snus, and naswar). Combusted products generate numerous carcinogens, particularly tobacco-specific nitrosamines (TSNAs) such as N-nitrosonornicotine (NNN) and 4-(methylnitrosamino)-1-(3-pyridyl)-1-butanone (NNK), which are excreted in urine and directly contact the bladder epithelium [22]. These compounds induce DNA adduct formation, oxidative stress, and apoptosis in urothelial cells, leading to epithelial barrier dysfunction. Tobacco smoke exposure also activates reactive oxygen species (ROS) generation and activates inflammatory cytokines such as IL-1β and IL-8, resulting in chronic mucosal inflammation and urothelial remodeling [23]. Smokeless tobacco users, although avoiding combustion, still experience sustained TSNA exposure and oxidative DNA damage, predisposing to urothelial dysplasia and inflammation [24].

Despite growing evidence linking lifestyle factors to bladder dysfunction, substantial knowledge gaps remain. Most research has focused on tobacco-related carcinogenesis, while the non-malignant, mechanistic interplay between dietary fat, tobacco exposure, and LUTS remains underexplored. Clarifying these interactions is crucial for understanding the metabolic-urothelial axis, the pathway through which systemic metabolic disturbances and environmental exposures converge on bladder health.

Therefore, this systematic review aims to synthesize mechanistic evidence on the impact of high-fat dietary intake and tobacco exposure, both smoked and smokeless, on urothelial integrity, bladder inflammation, and the development or progression of LUTD.

## Review

Methods

This systematic review was conducted in alignment with the Preferred Reporting Items for Systematic Reviews and Meta-Analyses (PRISMA) 2020 guidelines [25]. The review was designed to synthesize current evidence on the associations and mechanistic pathways through which high-fat dietary exposure and tobacco exposure influence urothelial biology, bladder inflammation, lower urinary tract symptoms, infection-related outcomes, and bladder carcinogenesis in human and relevant mammalian models. Established systematic review practices for both clinical/observational and preclinical evidence streams were integrated to ensure consistent appraisal and synthesis across study types. This review was not prospectively registered in PROSPERO.

Eligibility Criteria

The eligibility criteria were defined using a Population, Exposure, Comparator, Outcome, and Study Design (PECOS) framework tailored to the research question on dietary fat and tobacco exposures and bladder/urothelial outcomes [26,27].

Population: The population included human adults aged 18 years or older of any sex and animal models commonly used in mechanistic or translational bladder research, including rodents such as mice, rats, and rabbits, non-human primates, and other vertebrates, provided the models were relevant to urothelial biology or bladder inflammation. Pediatric human studies and non-relevant species, including invertebrate models not applicable to mammalian bladder biology, were excluded. Studies relying solely on cancer cell lines without a clear linkage to the specified exposures were also excluded.

Exposure: The exposures of interest comprised high-fat dietary exposure and tobacco exposure. For this review, high-fat dietary exposure was operationally defined as diets explicitly identified by the original investigators as high-fat, fat-enriched, or having a higher fat composition than the comparator/control diet. In animal studies, this was typically based on controlled feeding regimens with specified diet composition and exposure duration, while in human studies, dietary fat exposure was primarily assessed using food frequency questionnaires or dietary pattern assessment. Tobacco exposure was operationally defined as active or passive exposure to combusted tobacco products, smokeless tobacco products, or tobacco-related constituents such as nicotine and tobacco smoke, as reported in the included studies. Exposure assessment and reporting varied across studies and included self-report questionnaires, medical records, biomarker validation, and experimentally controlled exposure protocols. Studies describing a “Western diet” without defining high-fat content were excluded. Non-tobacco inhalants, including marijuana and non-tobacco-derived vaping products, and environmental toxins unrelated to tobacco were not considered eligible exposures.

Comparator: Eligible comparators included non-exposed, unexposed, or relatively lower-exposure groups, as defined by the original studies. In human studies, this included participants on standard or lower-fat diets and never-smokers, non-users, or reference groups with lower tobacco exposure, where applicable. In animal studies, comparators included standard diet controls, sham-exposed groups, vehicle controls, or lower-intensity exposure groups when these were explicitly defined by the original investigators. Studies lacking a clear comparator were excluded, where this prevented meaningful interpretation of exposure-outcome relationships.

Outcome: To be included, studies were required to report at least one bladder, urothelial, or LUTS-related outcome. Relevant human outcomes included storage, voiding, and post-micturition symptom domains. Outcomes applicable to human or animal studies encompassed measures of bladder health and urothelial integrity, such as tight junction protein expression, urothelial permeability and barrier disruption, epithelial thinning, inflammatory and immune markers, oxidative stress, apoptosis, fibrosis, and microbiome alterations affecting urothelial function. Clinical and infection-related outcomes included cystitis, recurrent urinary tract infection, and bladder inflammation. Oncologic outcomes included urothelial carcinoma incidence, progression, dysplasia, and prognostic or survival data when reported. Mechanistic outcomes clarifying pathways involving oxidative stress and DNA damage, immune activation and cytokine signaling, neurogenic inflammation, metabolic or endocrine disruptions, and carcinogenic mechanisms linked to tobacco toxins were eligible and synthesized to support biological plausibility.

Study design: The study designs eligible for inclusion comprised observational human studies, including cross-sectional, case-control, and cohort designs, interventional human studies evaluating diet modification or tobacco cessation, and human tissue-based mechanistic studies using biopsies, explants, or urothelial cell assays with linkage to exposure status. Eligible animal studies included controlled feeding studies, smoke or tobacco-exposure experiments, mechanistic bladder and urothelial investigations, and toxicology or carcinogenesis models relevant to bladder outcomes. Mechanistic experimental studies were included when they clarified biological pathways linking exposures to outcomes. Reviews, editorials, commentaries, letters, conference abstracts, and case reports or case series lacking adequate methodological quality were excluded, as were studies judged to have major methodological flaws or critically high risk of bias. Only English-language primary research articles published between January 2012 and December 2025 were eligible, and grey literature was considered selectively to explore publication bias and identify potentially relevant unpublished data. Duplicate publications were screened, and the most complete version was retained.

Information Sources and Search Strategy

A comprehensive literature search was conducted in PubMed/MEDLINE, the Cochrane Library, Scopus, Web of Science, and Google Scholar. The search aimed to capture contemporary evidence relevant to high-fat diet and tobacco-related bladder outcomes across both clinical and preclinical domains. The search strategy combined controlled vocabulary and free-text terms, and used Boolean operators to link exposure and outcome concepts. Core exposure terms included high-fat diet, dietary fat, fat-rich diet, Western diet, obesity, insulin resistance, metabolic syndrome, tobacco smoking, tobacco product, smokeless tobacco, and nicotine. These were combined with outcome terms including lower urinary tract symptoms, LUTS, cystitis, bladder inflammation, urothelium, urothelial permeability, tight junction proteins, and bladder cancer.

Study Selection and Screening

The selection process followed PRISMA guidance and was conducted in two stages. After removal of duplicates in EndNote 2021 (Clarivate, London, UK) [28], two independent reviewers screened titles and abstracts to identify studies potentially meeting eligibility criteria, excluding records that were clearly irrelevant to high-fat dietary exposure, tobacco exposure, or bladder/urothelial outcomes. Full-text articles of all potentially eligible studies were then retrieved and assessed independently by the same reviewers against the predefined criteria. Disagreements were resolved through discussion, and a third reviewer was consulted when consensus could not be reached. No automation tools were used at any stage of the selection process. A PRISMA flow diagram was constructed to document the number of records identified, duplicates removed, records excluded during title and abstract screening, full-text articles excluded, and the final number of studies included.

Data Extraction and Quality Assessment

Data were extracted using a standardized data collection form. The form was piloted on a subset of included studies to ensure consistency and completeness of captured variables. Extracted data included author and year, model, study design, population, exposures, and summary.

Risk of Bias

Risk of bias was assessed independently by two reviewers using tools appropriate to the study design. Human studies (which were primarily non-randomized/observational) were assessed using the Risk Of Bias In Non-randomized Studies of Interventions (ROBINS-I), evaluating bias due to confounding, selection of participants, classification of exposures, deviations from intended exposures, missing data, measurement of outcomes, and selection of the reported result [29]. Animal studies were assessed using the Systematic Review Centre for Laboratory Animal Experimentation (SYRCLE)’s risk of bias [30]. Risk-of-bias assessments were visualized using the Risk of Bias Visualization tool (ROBVIS) [31]. Judgments were resolved by consensus, with third-reviewer input when necessary.

Data Synthesis

A narrative synthesis was performed across human, animal, and mechanistic evidence streams, structured by exposure category and outcome domain to allow integrated interpretation of clinical associations and biological plausibility.

Results

Process of Identifying and Screening Studies

**Figure 1:**
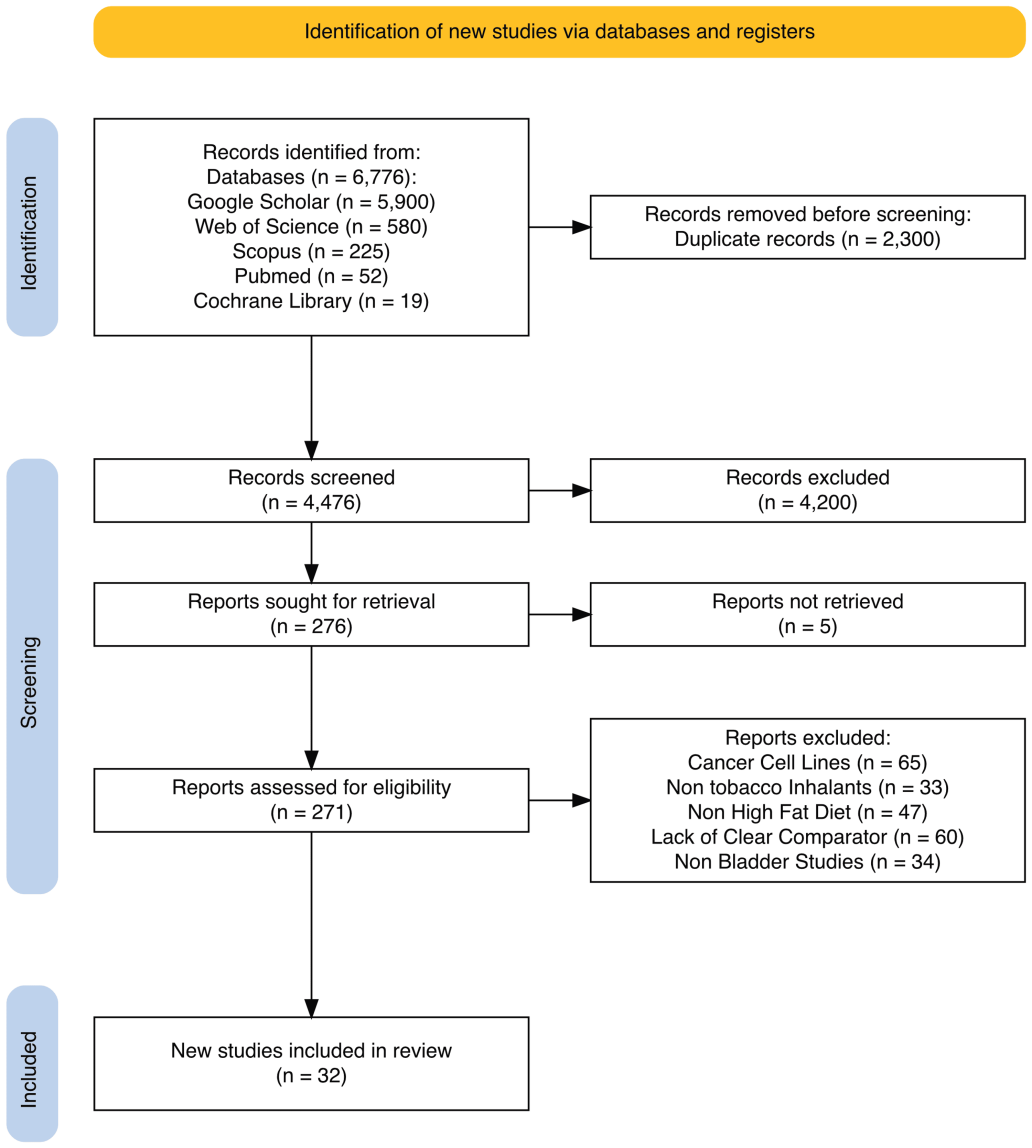
PRISMA flowchart of selected studies PRISMA: Preferred Reporting Items for Systematic Reviews and Meta-Analyses

A total of 32 studies met the eligibility criteria and were ultimately included in this review. In the initial search, 6,776 records were retrieved across multiple databases: 5,900 from Google Scholar, 580 from Web of Science, 225 from Scopus, 52 from PubMed, and 19 from the Cochrane Library. All duplicates were removed, and studies that did not meet the predefined inclusion criteria were excluded during the screening process. In accordance with PRISMA guidelines and using the PRISMA tool [32], a flowchart was constructed to illustrate the systematic and rigorous process through which studies were identified, screened, assessed for eligibility, and selected for inclusion (Figure 1).

Characteristics of the Included Studies

A total of 32 papers were reviewed in this study, all of which met our inclusion criteria. Across 19 studies, tobacco products were frequently associated with LUTS and increased risks of bladder cancer. Among 13 studies, HFD commonly resulted in altered detrusor muscle contractility, increased oxidative stress and inflammatory markers, urothelial dysplasia, decreased bladder compliance, and apoptosis. Studies also show that diet-induced obesity increases susceptibility to cystitis, fibrosis, and bladder cancer.

Table 1 summarizes findings from 14 human studies across various study designs and tobacco products. Most of which consistently showed association with worsened LUTS, impaired bladder function, and increased bladder risk. Some studies highlighted molecular and genetic effects of tobacco product exposure, such as oxidative stress, inflammatory signaling, changes in nitric oxide pathways, alterations in DNA methylation, and reduced detoxification of carcinogens. Diet-related evidence in humans was limited and was represented mainly by an observational study of dietary fat sources/quality rather than a strictly defined high-fat diet, with heterogeneous associations across different foods and bladder cancer risk. Reported symptoms included urgency, nocturia, and urinary incontinence (Table 1).

**Table 1:** Summary of human studies on tobacco products, high-fat diet, and their effects on bladder function KCHS: Korean Community Health Survey, LUTS: lower urinary tract symptoms, ETS: environmental tobacco smoke, icNO: intracellular nitric oxide, NO: nitric oxide, GAG: glycosaminoglycan, WBC: white blood cell, GSTM1: glutathione S-transferase mu 1, NAT2: N-acetyltransferase 2, WGCNA: weighted gene co-expression network analysis, KIR2DL5: killer cell immunoglobulin-like receptor 2DL5, PVR: poliovirus receptor, e-cigarette: electronic cigarette, FFQ: Food Frequency Questionnaire, NHANES: National Health and Nutrition Examination Survey

S/N	Reference	Sex	Study design	Population	Exposure/composition	Key findings
1	Noh et al. (2020) [33]	Male	Cross-sectional analysis using KCHS	86,707 adults (≥19 years)	Cigarette smoking	Impairs bladder blood flow, increasing oxidative stress, urothelial irritation, and increased androgen levels
						Alters autonomic balance, enhancing sympathetic tone and impaired parasympathetic-mediated voiding
						Inflammatory cytokines, bladder wall irritation, and fibrosis
2	Ozden et al. (2025) [34]	Male	Observational study	50 patients	Chronic tobacco (maras powder) use	Direct urothelial toxicity
						Cholinergic overstimulation and long-term receptor desensitization detrusor instability
						High oxidative stress and inflammatory infiltration degrade GAG layers, increasing bladder permeability/leaky urothelium
3	Hajjar et al. (2022) [35]	Nulliparous female	Cross-sectional questionnaire-based survey	767 university students	Waterpipe smoking, cigarette use	Higher urgency, urinary incontinence
4	Choo et al. (2015) [36]	Male (elderly men)	Prospective longitudinal cohort study	224 participants	Cigarette smoking	Cigarette smoking (among other exosomic factors) poses as a risk factor for LUTS development/progression over time
5	Kawahara et al. (2020) [37]	Male (Japanese men)	Cross-sectional population-based study using a web survey questionnaire	9,042 participants	Cigarette smoking	The effect was more marked among younger men than older ones
6	Yang et al. (2025) [38]	Male and female	Cross-sectional analysis of a large national survey (NHANES)	NHANES 2005-2020 participants: 43,372 (≥20 years), NHANES 2015-2018 participants (≥20 years): 11,283 (e-cigarette), 8,402 (dual use)	Cigarette use, e-cigarette use, and dual use (cigarette + e-cigarette)	Cigarette, e-cigarette, and dual use could lead to nocturia and urge urinary incontinence
7	Bolat et al. (2015) [39]	Male	Retrospective observational study	186 patients	Cigarette smoking	Chronic smoking impairs bladder outflow/detrusor contractility
						Reduced lung function reflects systemic effects of smoking
						This could also impair pelvic floor or detrusor muscle function
8	Shi et al. (2024) [40]	Male and female	Cross-sectional analysis using data	10,195 adults	Tobacco smoke (active and passive)	Chronic exposure to tobacco smoke components may increase low-grade inflammation in the bladder or pelvic floor muscles and weaken urethral closure, thereby increasing propensity to leak; even passive exposure (ETS) may deliver enough toxins to disrupt bladder control mechanisms
9	Ruiz-Lorente et al. (2025) [41]	Male	Case-control genetic/immunologic study	2,115 healthy Caucasian men	icNO from tobacco smoking	KIR2DL5 interacts with the PVR on bladder cells, suppressing immune cell activation
						Simultaneously, changes in NO levels may create an environment that supports tumor growth
10	Vermeulen et al. (2024) [42]	Male and female	Nested case-control study	789 cases	Smoking-related DNA methylation markers in white blood cells	Methylation patterns in WBC likely serve as biomarkers of cumulative biological exposure to tobacco smoke
11	Avirmed et al. (2021) [43]	Male and female	Hospital-based case-control genetic epidemiology study	120 patients	Tobacco smoking	GSTM1 and NAT2 are enzymes involved in the metabolic detoxification of tobacco-related carcinogens
						Absence of GSTM1 and slow acetylation of NAT2 accumulate activated carcinogenic metabolites in the urothelial DNA and promote bladder carcinogenesis
12	Gao et al. (2023) [44]	Not reported	Combined bioinformatic and genetic association study	1,681 cases (Chinese population), 11,398 cases (European population)	Tobacco smoking	Tobacco smoking increases the susceptibility to bladder cancer
			Application of WGCNA on transcriptome data of bladder cancer tissues			
13	Kwan et al. (2022) [45]	Male	Prospective cohort study	1,472 adults	Tobacco use (cigarettes)	Heavy/long-term cigarette smoking is associated with a higher risk of recurrence of bladder cancer
14	Teng et al. (2023) [46]	Male and female	Observational case-control, dietary assessment for a duration of one year	405 participants	Intake of fatty foods; cooking oil (soybean oil), red meat, marine fish, eggs, milk/dairy products, nuts	The study suggests that soybean oil may increase bladder cancer risk, while consumption of fish, nuts, and dairy may be protective

This review also compiles findings from 18 preclinical studies conducted on rodent and rabbit models (Table 2). The studies consistently showed that a high-fat diet led to bladder dysfunction, characterized by oxidative stress, inflammation, fibrosis, impaired detrusor contractility, altered bladder compliance, and urothelial injury. On the other hand, tobacco-related exposures were found to also cause urothelial hyperplasia, DNA damage, and increased susceptibility to carcinogenesis. Collectively, these models demonstrate common pathways of bladder injury driven by metabolic stress and tobacco exposure.

**Table 2:** Summary of animal studies on tobacco products, high-fat diet, and their effects on bladder function HFD: high-fat diet, HFS: high-fat/high-sucrose, OH-BBN: N-butyl-N-(4-hydroxybutyl)nitrosamine, eNOS: endothelial nitric oxide synthase, RhoA/ROCK: Ras homolog family member A/Rho-associated protein kinase, TNF-α: tumor necrosis factor alpha, IGF-1: insulin-like growth factor 1, ECS: electronic cigarette smoke, TS: tobacco smoke, EMT: epithelial-mesenchymal transition

S/N	Reference	Model	Study design	Population	Exposure/composition	Key findings
1	Xu et al. (2023) [47]	Male Harlan-Sprague Dawley mice	Preclinical; mice exposed to a carcinogen to induce bladder carcinogenesis for 12 weeks	120 mice (post-survival)	OH-BBN-induced bladder carcinogenesis model (40 weeks)	Tobacco-smoking related bladder cancer might be mitigated by kawain, which counteracts smoking-induced epigenetic dysregulation
2	Morelli et al. (2012) [48]	Male New Zealand white rabbits	Preclinical	65 rabbits	HFD	Bladder dysfunction, RhoA/ROCK overactivity in bladder smooth muscle
3	Wada et al. (2023) [49]	Female Sprague-Dawley rats	Preclinical 12-week-old/12 weeks	33 rats	HFS diet	HFS diet-induced systemic changes (muscle loss, metabolic stress) may contribute to lower urinary tract dysfunction
4	Oberbach et al. (2013) [50]	Male Sprague-Dawley rats	Preclinical four-week-old or HFD groups for 11 weeks	30 rats	HFD	HFD may lead to bladder dysfunction via hypoxia, oxidative stress, impaired smooth muscle signaling, decreased eNOS, and fibrosis
						All these contributing to impaired contractility, altered bladder compliance, and incomplete bladder emptying
5	Aizawa et al. (2013) [51]	Male; C57BL/6J mice	Mice were assigned to either a normal diet or an HFD for 20 weeks	32 mice	HFD	Despite metabolic changes, HFD-feeding for 20 weeks affects bladder functioning
6	Wu et al. (2023) [52]	Male Sprague-Dawley rats	Preclinical for 42 weeks	90 rats	60% fructose diet	The study shows a link between diet-induced metabolic disturbances and bladder dysfunction; however, this is determined by the nature of the HFD
					2% cholesterol + 10% lard diet	
					30% fructose + 20% lard diet	
					32.5% lard diet	
7	de Souza et al. (2019) [53]	Rat (sex not reported)	Preclinical 60-day-old rats (120 days)	60 rats	HFD (lard-based) + Brazil nut oil	HFD impairs bladder contractility and function; supplementation with Brazil nut oil attenuates some epithelial but not all collagen changes
8	Fan et al., 2014 [54]	Male C57BL/6J (B6) mice	Preclinical eight-week-old mice	24 mice	HFD	HFD-induced systemic inflammation (TNF-α) alters bladder smooth muscle contractility
			12 weeks or antagonist/etanercept (last four weeks)		HFD + vehicle, (iii) HFD + antagonist treatment; high-fat diet was given for 12 weeks; antagonist, etanercept, was administered the last four weeks	
9	Kanpalta Mustafaoğlu et al. (2022) [55]	Male Wistar rats	Preclinical 16 weeks	24 rats	HFD	HFD leads to bladder damage via oxidative stress and inflammation
					HFD + plant extract treatment groups	
10	de Andrade et al. (2022) [56]	Female C57BL/6J mice	Preclinical cystitis model, seven-week-old	16 mice	HFD for eight weeks	Obesity induced by an HFD promotes increased urothelial susceptibility to inflammation, proliferation, apoptosis, and dysplasia
						This suggests an exacerbation of cystitis and long-term risk for bladder cancer
11	Kanpalta Mustafaoğlu et al. (2025) [57]	Male Wistar albino rats	Preclinical 16 weeks	32 rats	HFD	HFD induces bladder injury by shedding of apical urothelial cells, vascular congestion, and increased collagen deposition in the muscular layer
12	Adedeji and Olapade-Olaopa (2018) [58]	Male Wistar albino rats	12 weeks of exposure to HFD with unobstructed and obstructed bladder groups	80 rats	HFD; 22% protein, 13.5% carbohydrates, 60% fat, and 4.5% crude fiber	HFD increased bladder weight, bladder inflammation, and collagen content, subjecting the bladder to decreased compliance and urothelial integrity; overall, it induced fibrosis
13	Adedeji and Olapade-Olaopa (2021) [59]	Male Wistar albino rats	12 weeks of exposure to HFD with unobstructed and obstructed bladder groups	80 rats	HFD; 22% protein, 13.5% carbohydrates, 60% fat, and 4.5% crude fiber	HFD decreased detrusor contractile response and increased IGF-1 in the bladder, which poses high risks of bladder cancer (promoting cell growth and inhibiting death)
14	Suzuki et al. (2018) [60]	Male rats	Preclinical F344 rat bladder carcinogenesis (four-week study)	175 males (six weeks old)	Nicotine (drinking water; 32 weeks)	Oral nicotine promotes urinary bladder carcinogenesis in rats, increasing proliferation and leading to hyperplasia
15	Tang et al. (2019) [61]	Male FVB/N mice	Long-term exposure (54 weeks) to ECS versus controls (vehicle/filtered air)	85 mice	ECS, nicotine	Long-term exposure to ECS induces hyperplastic lesions and DNA damage in the bladder urothelium of mice
16	Dodmane et al. (2014) [62]	Female Wistar Han rats and female C57Bl/6 mice	Short-term (four-week) oral toxicity study	20 rats	Nicotine in drinking water	Nicotine induces urothelial hyperplasia in both rats and mice
				20 mice		
17	Wang et al. (2017) [23]	Male BALB/c mice	In vivo experimental; mice were exposed to TS for 24 weeks	36 mice (6-8 weeks old)	TS	TS induces urothelial hyperplasia and EMT in mice bladder, linked to pre-neoplasia and cancer progression
18	Liang et al. (2015) [63]	Male BALB/c mice	In vivo experiment; mice were exposed to TS for 24 weeks, with some groups receiving a chemopreventive agent (curcumin)	30 mice (at eight weeks)	TS	TS induces precancerous molecular changes in the bladder

Table 3 presents the methods used to measure key exposure in the included studies. Tobacco exposure was assessed through self-reported questionnaires, medical records, biomarker validation (such as cotinine and methylation markers), and controlled experimental protocols in animal models. Categories of exposure included combusted tobacco, smokeless tobacco, electronic cigarettes, nicotine, and passive smoke. High-fat diet exposure was measured using controlled feeding regimens in animal studies, while food frequency questionnaires were used in human studies, with comparator groups consistently receiving standard diets/non-smokers.

**Table 3:** Exposure categories and how they were measured PECOS: Population, Exposure, Comparator, Outcome, and Study Design, ECS: electronic cigarette smoke, HFD: high-fat diet, FFQ: Food Frequency Questionnaire

S/N	Reference	Exposure category (PECOS “E”)	Exposure measurement method (as reported)	Comparator
1	[33,36,37,39,41-45]	Cigarette smoking (combusted)	Self-report questionnaires, medical records, baseline smoking history, and methylation markers	Never-smokers/non-smokers/healthy controls
2	[35]	Waterpipe	Self-administered questionnaire	Control
3	[34]	Smokeless tobacco	Interview + medical records	Control
4	[23,40,63]	Active and/or passive tobacco smoke	Serum cotinine + questionnaire, controlled tobacco smoke protocol	Control
5	[47]	Tobacco carcinogen	Epigenetic marker (methylation)	Vehicle diet with no bladder carcinogen
6	[38,61]	ECS	Self-report survey, controlled ECS exposure	Non-users, filtered air
7	[60,62]	Nicotine	Nicotine in drinking water	Tap water, control
8	[48,50,51,53-59]	HFD	Controlled feeding; diet composition specified variably	Standard, regular diet
9	[49,52]	High-fat/high-sucrose and high-fructose diet	Controlled feeding; multiple diet arms	Standard, regular diet
10	[46]	Dietary fat	FFQ (fatty foods, cooking oil types, and animal foods)	Control

The distribution of outcome domains assessed across included studies, as seen below in Table 4, reported that the human studies were related to storage and voiding bladder dysfunctions, while the animal studies primarily evaluated bladder capacity, compliance, detrusor contractility, and histopathological alterations.

**Table 4:** Outcome domain map aligned to LUTS, urothelial integrity, inflammation, and carcinogenesis PECOS: Population, Exposure, Comparator, Outcome, and Study Design, LUTS: lower urinary tract symptoms, UUI: urge urinary incontinence, OAB: overactive bladder, NF-κB: nuclear factor kappa B, IKKβ: IκB kinase beta, ICAM-1: intercellular adhesion molecule 1, CRP: C-reactive protein, CXCL12: C-X-C motif chemokine ligand 12, IGF-1: insulin-like growth factor 1, NGF: nerve growth factor, HIF-1α: hypoxia-inducible factor 1 alpha, 8-OHdG: 8-hydroxy-2′-deoxyguanosine, MDA: malondialdehyde, MPO: myeloperoxidase, ROS: reactive oxygen species, eNOS: endothelial nitric oxide synthase, ECM: extracellular matrix, EMT: epithelial-mesenchymal transition

S/N	Reference	Outcome domain (PECOS “O”)	Damage
1	[33,35-38,40]	LUTS - storage symptoms	Urgency, frequency, nocturia, UUI/OAB-type symptoms
2	[33,37,39]	LUTS - voiding symptoms, emptying	Weak stream, hesitancy, incomplete emptying, poor flow
3	[34,49,51,52,58]	Bladder capacity, compliance (functional)	Capacity, compliance, voided volume, cystometry/urodynamics
4	[48,49,51,53,54,59]	Detrusor contractility, smooth muscle signaling	Bladder strip contractility, muscarinic responses, neural stimulation responses
5	[23,34,53,56-59,61-63]	Urothelial integrity and histopathology	Erosion, hyperplasia, dysplasia, epithelial thickness, mucosal damage
6	[23,33,34,54-59,63]	Inflammation, immune activation	Cytokine-related pathways, immune infiltration, NF-κB/IKKβ, ICAM-1, CRP, CXCL12, IGF-1, NGF
7	[33,34,49,50,55-58,61,62]	Oxidative stress, DNA damage	HIF-1α, 8-OHdG, MDA, MPO, ROS-associated changes, DNA damage endpoints
8	[49,50,58]	Hypoxia, vascular impairment	HIF-1α, eNOS signaling, ischemic remodeling proxies
9	[49,50,53,57-59]	Fibrosis, ECM remodeling	Collagen deposition, ECM accumulation, wall remodeling
10	[23,41-47,56,59-63]	Carcinogenesis, precancer	Urothelial lesions, EMT markers, proliferative signaling, bladder cancer risk/recurrence

Risk of Bias Assessment

**Figure 2:**
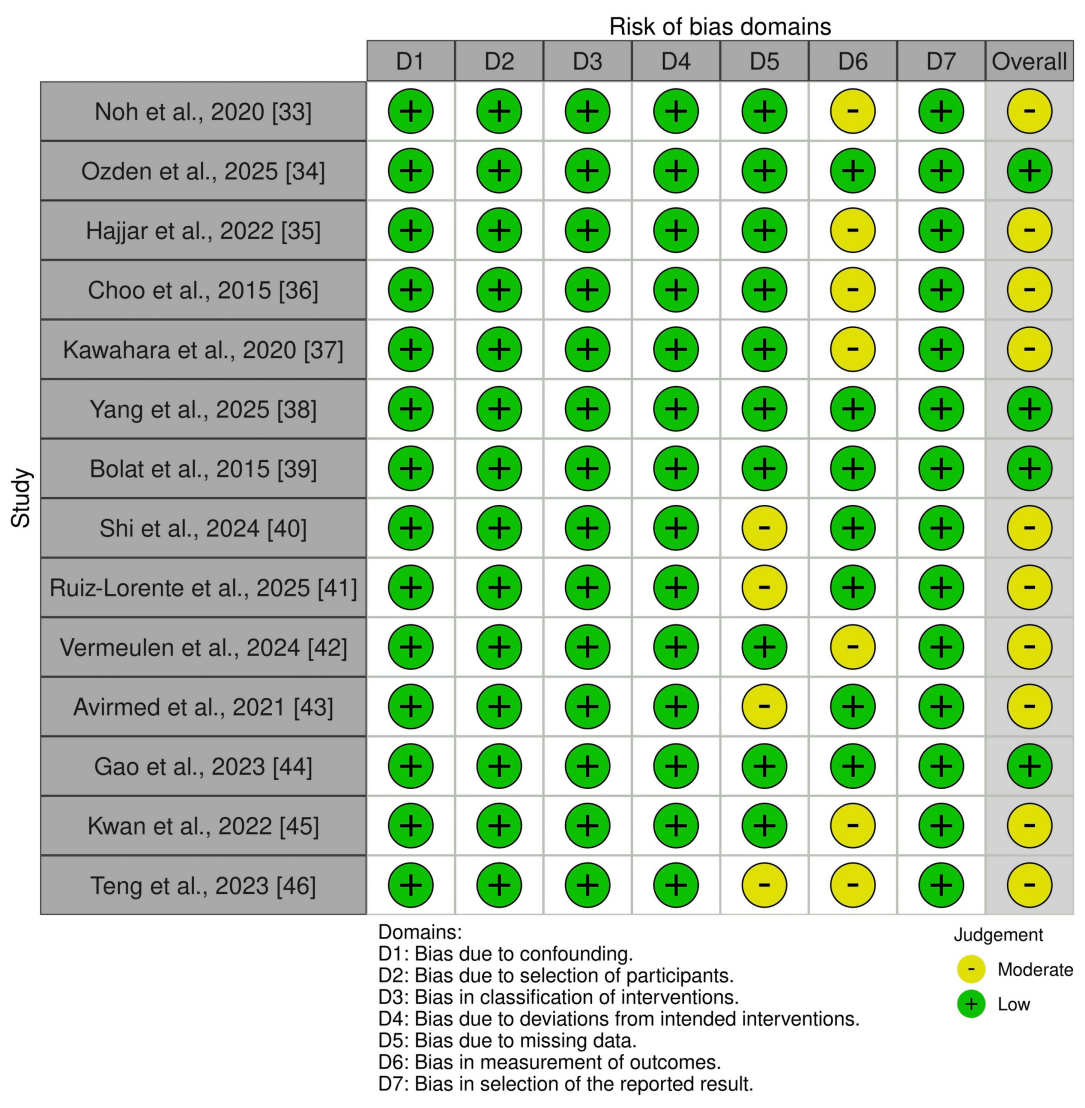
Risk of bias using the ROBINS-I tool for human studies ROBINS-I: Risk Of Bias In Non-randomized Studies of Interventions

**Figure 3:**
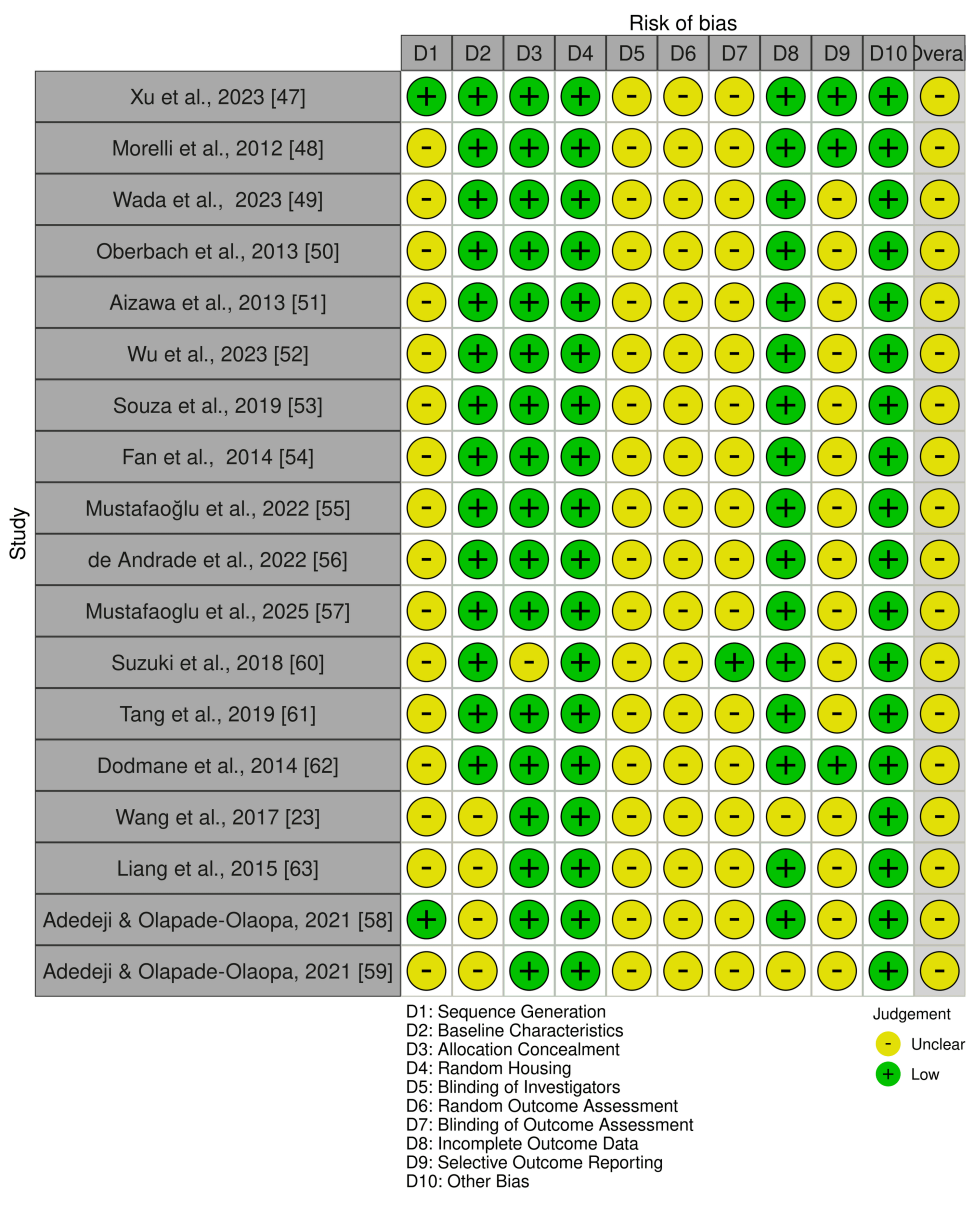
Risk of bias using the SYRCLE tool for animal studies SYRCLE: Systematic Review Centre for Laboratory Animal Experimentation

Risk of bias was assessed separately for human and animal studies using study design-specific tools. Human studies were evaluated using the ROBINS-I tool, while animal studies were assessed using SYRCLE’s Risk of Bias tool [29,30]. The detailed domain-level assessments are presented in Figure 2 and Figure 3, with risk-of-bias judgments from both tools visualized using the Risk of Bias Visualization tool (ROBVIS) [31].

Risk of bias for human studies (Figure 2) was generally low across most domains. All studies were rated as low risk for bias due to confounding (D1), selection of participants (D2), classification of exposures (D3), deviations from intended exposures (D4), and selection of the reported result (D7). Moderate risk was observed primarily in the domains of missing data (D5) and measurement of outcomes (D6). Overall, 10 studies were judged as having a moderate risk of bias, while the remaining studies were rated as low overall risk.

Furthermore, the animal studies assessed (Figure 3) demonstrated some unclear judgments across sequence generation (D1), blinding-related (D5-D7), and selective reporting (D9) domains. Allocation concealment (D3), random housing (D4), and incomplete outcome data (D8) were more commonly judged as low risk. All animal studies were rated as having an overall unclear risk of bias (Table 5, Figure 3).

**Table 5:** Summary of SYRCLE risk of bias for preclinical included studies on tobacco products, high-fat diet, and their effects on bladder function SYRCLE weight quantity scores: low = 1, unclear = 0.5, high = 0 SYRCLE: Systematic Review Centre for Laboratory Animal Experimentation

S/N	Reference	Sequence generation	Baseline characteristics	Allocation concealment	Random housing	Blinding of investigators	Random outcome assessment	Blinding of outcome assessment	Incomplete outcome data	Selective outcome reporting	Other bias	Overall	Weight (%)
1	Xu et al. (2023) [47]	Unclear	Unclear	Low	Low	Unclear	Unclear	Unclear	Low	Unclear	Low	Unclear	7.5
2	Morelli et al. (2012) [48]	Low	Low	Low	Low	Unclear	Unclear	Unclear	Low	Unclear	Low	Unclear	8.5
3	Wada et al. (2023) [49]	Unclear	Low	Low	Low	Unclear	Unclear	Unclear	Low	Unclear	Low	Unclear	8
4	Oberbach et al. (2013) [50]	Unclear	Low	Low	Low	Unclear	Unclear	Unclear	Low	Unclear	Low	Unclear	8
5	Aizawa et al. (2013) [51]	Unclear	Low	Low	Low	Unclear	Unclear	Unclear	Low	Unclear	Low	Unclear	8
6	Wu et al. (2023) [52]	Unclear	Low	Low	Low	Unclear	Unclear	Unclear	Low	Unclear	Low	Unclear	8
7	de Souza et al. (2019) [53]	Unclear	Low	Low	Low	Unclear	Unclear	Unclear	Low	Unclear	Low	Unclear	8
8	Fan et al. (2014) [54]	Unclear	Low	Low	Low	Unclear	Unclear	Unclear	Low	Unclear	Low	Unclear	8
9	Kanpalta Mustafaoğlu et al. (2022) [55]	Unclear	Low	Low	Low	Unclear	Unclear	Unclear	Low	Unclear	Low	Unclear	8
10	de Andrade et al. (2022) [56]	Unclear	Low	Low	Low	Unclear	Unclear	Unclear	Low	Unclear	Low	Unclear	8
11	Kanpalta Mustafaoğlu et al. (2025) [57]	Unclear	Low	Low	Low	Unclear	Unclear	Unclear	Low	Unclear	Low	Unclear	8
12	Suzuki et al. (2018) [60]	Unclear	Low	Low	Low	Unclear	Unclear	Unclear	Low	Unclear	Low	Unclear	8
13	Tang et al. (2019) [61]	Unclear	Low	Unclear	Low	Unclear	Unclear	Low	Low	Unclear	Low	Unclear	7
14	Dodmane et al. (2014) [62]	Unclear	Low	Low	Low	Unclear	Unclear	Unclear	Low	Low	Low	Unclear	8
15	Wang et al. (2017) [23]	Unclear	Low	Low	Low	Unclear	Unclear	Unclear	Low	Low	Low	Unclear	8.5
16	Liang et al. (2015) [63]	Unclear	Unclear	Low	Low	Unclear	Unclear	Unclear	Unclear	Unclear	Low	Unclear	7
17	Adedeji and Olapade-Olaopa (2021) [58]	Low	Unclear	Low	Low	Unclear	Unclear	Unclear	Low	Unclear	Low	Unclear	8
18	Adedeji and Olapade-Olaopa (2021) [59]	Unclear	Unclear	Low	Low	Unclear	Unclear	Unclear	Unclear	Unclear	Low	Unclear	7

Discussion

The evidence gathered in this systematic review consistently supports that both high-fat dietary intake and tobacco exposure adversely affect bladder health through convergent biological mechanisms and correlate with worse LUTS and bladder pathology. Across a spectrum of human epidemiological studies, clinical cohorts, and mechanistic experiments in animal models, these lifestyle exposures emerge as significant modifiers of urothelial integrity and bladder function. Chronic HFD consumption and tobacco use, whether through cigarettes, waterpipe, or smokeless products, induce oxidative stress, inflammatory signaling, and tissue remodeling within the bladder that can manifest as both functional disturbances (storage and voiding LUTS), structural damage (urothelial barrier dysfunction and fibrosis), and even malignant transformation.

Included studies examining high-fat diet exposure demonstrate that chronic lipid excess induces a state of metabolic inflammation and oxidative stress that directly affects bladder tissue. Experimental models repeatedly show that high-fat feeding compromises urothelial barrier integrity through altered tight junction protein expression and increased epithelial permeability, facilitating inflammatory cell infiltration and sustained mucosal irritation [64]. These urothelial changes are accompanied by increased proliferative activity, apoptotic turnover, and activation of canonical inflammatory pathways such as NF-κB, creating a microenvironment characterized by chronic injury and repair. Importantly, these molecular and cellular alterations are not isolated but occur with functional disturbances, including altered voiding patterns and impaired detrusor contractility, supporting a link between metabolic stress and LUTS development [56].

Moreover, a recurring theme across included preclinical studies is the induction of bladder hypoxia and oxidative DNA damage in response to high-fat feeding. Upregulation of hypoxia-inducible factors and accumulation of oxidative DNA lesions suggest that impaired microvascular perfusion is a key mediator of diet-induced bladder injury [50]. This interpretation is reinforced by evidence of endothelial dysfunction, reduced nitric oxide bioavailability, and downregulation of endothelial nitric oxide synthase within the bladder wall. Given the essential role of nitric oxide in maintaining smooth muscle relaxation, vascular tone, and urothelial defense, its reduction provides a unifying explanation for the emergence of chronic ischemia, inflammation, and subsequent fibrotic remodeling observed in multiple models [49,65]. Consistent with this, diet-obstruction interaction evidence indicates that HFD can amplify bladder injury rather than simply co-occur with LUTD, intensifying inflammatory and pro-remodeling signaling along a hypoxia-fibrosis trajectory that undermines compliance and urothelial stability [58]. Importantly, the diet-reversal model suggests these early HFD-linked changes may be partly reversible with dietary correction, reinforcing lifestyle intervention as a mechanistically justified adjunct to LUTD/LUTS management [59].

Another key pathological change induced by high-fat feeding is extracellular matrix remodeling and fibrosis in the bladder, demonstrated in multiple rodent studies that met our inclusion criteria. There were consistent reports of increased collagen deposition, smooth muscle hypertrophy, bladder wall thickening, and reduced compliance, changes that are well-recognized contributors to both storage and voiding symptoms. The involvement of profibrotic signaling pathways, including TGF-β and RhoA/ROCK activation, places bladder remodeling within a broader framework of obesity-associated organ fibrosis [48,53]. Observed partial reversal of these changes by antioxidant interventions supports a causal role for oxidative stress and lipid peroxidation, and further highlights the potential reversibility of early diet-induced bladder injury [53].

In addition to histological changes, HFD has functional consequences on detrusor contractility and voiding behavior. However, results have varied, indicating a complex relationship between metabolic state and bladder neuromuscular function. Functional outcomes across high-fat diet models show some heterogeneity, with reports of bladder overactivity, underactivity, or minimal baseline dysfunction depending on species, diet composition, and exposure duration. This variability likely reflects different stages during the pathophysiology. Early metabolic stress may provoke irritative, overactive bladder phenotypes driven by inflammation and sensory nerve sensitization, whereas prolonged exposure appears to favor decompensated states marked by fibrosis, neuropathy, and reduced contractility [51,52,54]. This proposed trajectory mirrors patterns observed in diabetic bladder dysfunction and aligns with the consistent detection of molecular stress markers even in the absence of functional changes. These suggest that high-fat diet exposure initiates bladder pathology well before clinical symptoms fully manifest [6,66,67].

Human studies, while fewer in number, corroborate these experimental findings through strong associations between obesity, metabolic syndrome, and LUTS severity. Obesity is consistently identified as an independent risk factor for overactive bladder and urinary incontinence, with symptom improvement observed following modest weight loss. These clinical improvements reinforce the notion that metabolic stress contributes causally to bladder dysfunction rather than simply coexisting with it [35]. Emerging epidemiological evidence further suggests that dietary fat quality may influence bladder disease risk, including malignancy, with unsaturated fats appearing protective and certain fat sources associated with increased cancer risk [46,68,69]. These observations support a model in which systemic metabolic and inflammatory states, shaped by long-term dietary patterns, exert downstream effects on bladder biology [70,71].

The impact of tobacco exposure on bladder health extends across malignant and non-malignant disease spectrums. Tobacco smoke delivers a complex mixture of carcinogens and oxidants that are absorbed systemically and concentrated in urine, resulting in prolonged direct contact with the urothelium [21,72]. Experimental studies demonstrate that nicotine and other tobacco constituents induce urothelial injury, compensatory hyperplasia, oxidative stress, and dysregulated differentiation [60,62]. Moreover, tobacco exposure exerts measurable effects on bladder function and LUTS, as animal models meeting our inclusion criteria reveal that cigarette smoke, e-cigarette aerosol, and smokeless tobacco all induce urothelial hyperplasia, inflammation, fibrosis, and reduced bladder capacity, challenging the perception that non-combustible tobacco products are benign [61,73]. Furthermore, human epidemiological studies consistently link smoking with increased LUTS severity, particularly storage symptoms, reduced urinary flow, and higher residual volumes [33,36].

At the molecular level, tobacco exposure interacts with genetic susceptibility, epigenetic regulation, and immune surveillance. Emerging evidence also suggests that smoking alters immune signaling and may suppress anti-tumor surveillance mechanisms, adding complexity to its role in bladder pathology [41,42,44].

Although high-fat diet and tobacco exposure independently exert harmful effects on the bladder, they often coexist as lifestyle factors and may synergistically amplify bladder dysfunction. Epidemiologically, smokers may have poorer diets on average, and the combination of smoking and obesity is commonly seen in patients with metabolic syndrome [74]. Mechanistically, both exposures share overlapping pathogenic pathways: chronic inflammation, oxidative stress, endothelial dysfunction, and neurohumoral alterations. When combined, these factors could have more-than-additive effects on the lower urinary tract. For instance, both smoking and HFD promote systemic oxidative stress, smoking via ROS in smoke and inflammatory cell activation, and HFD via excess lipids inducing ROS in adipose tissue and the vasculature [50,75]. Together, a smoker on a high-fat diet would likely have an especially high burden of oxidative stress. This could overwhelm local antioxidant defenses in the bladder and lead to accelerated damage to urothelial cells and bladder nerve fibers. Nicotine may exacerbate diet-induced metabolic inflammation. Experimental studies have shown that nicotine can worsen insulin resistance by inducing mitochondrial stress and ceramide accumulation in adipocytes [75,76]. Nicotine also lowers adiponectin (an anti-inflammatory adipokine) and raises levels of circulating inflammatory cytokines [76]. In a high-fat-fed individual, whose adipose tissue may already be inflamed, smoking could further tilt the balance toward a pro-inflammatory state (higher TNF-α, IL-6, etc.), thereby magnifying the metabolic-urothelial axis of inflammation that injures the bladder.

Furthermore, both exposures activate the NF-κB pathway in tissues, HFD through TNF-α and nutrient-sensing pathways, and smoking through ROS and cytokine release. NF-κB drives transcription of many inflammatory mediators and fibrotic factors. When high-fat diet and smoking co-occur, NF-κB activation in the bladder may be especially robust and chronic, potentially leading to a vicious cycle of inflammation that underpins refractory OAB symptoms or painful bladder syndrome [56,77]. Additionally, these factors may together affect the bladder microbiome and urothelial innate immunity. There is emerging interest in how lifestyle influences the urinary microbiota. Smoking has been associated with changes in the urinary microbiome and an increased presence of bacteria that can modulate immune checkpoints [78].

This review underscores several important clinical implications for bladder health and the management of lower urinary tract symptoms [79]. Lifestyle modification should be a foundational element of care, as weight optimization and smoking cessation are supported by urologic evidence in addition to their general health benefits [80]. Weight loss may alleviate bladder symptoms by reducing abdominal pressure and systemic inflammation, while smoking cessation removes a direct urothelial irritant and may improve pelvic perfusion [81]. Notably, preclinical evidence further suggests that diet-associated bladder changes are not necessarily fixed. Dietary correction after high-fat exposure can partially reverse adverse bladder remodeling signals and functional impairment, supporting the clinical rationale for early dietary intervention [59]. These conservative measures can complement pharmacologic therapy, potentially decreasing reliance on invasive interventions. From a preventive perspective, the evidence reinforces public health messages that avoiding tobacco and maintaining a healthy diet substantially reduce bladder cancer risk, with clear dose-response relationships indicating that even partial exposure reductions may be beneficial [82]. Accordingly, routine assessment of diet and smoking status should be integrated into bladder health evaluations.

An important limitation of the present review is the relative imbalance between preclinical and human evidence for high-fat diet exposure. While animal studies consistently demonstrate mechanistic links between high-fat feeding and urothelial injury, inflammation, fibrosis, and bladder dysfunction, direct human evidence remains limited. Therefore, the human relevance of these findings should be interpreted with caution, and the current evidence is better viewed as supporting biological plausibility rather than definitive causality in humans.

The review also identifies key gaps that warrant further research. Human studies examining dietary fat patterns and LUTS are needed to validate animal findings, and longitudinal data could clarify whether dietary improvement leads to symptom reduction. Although the carcinogenic role of smoking is well established, more evidence is needed to determine whether smoking cessation consistently improves non-neurogenic LUTS over time. Potential sex differences in bladder responses to diet and tobacco exposure remain underexplored, as do the effects of combined lifestyle exposures that better reflect real-world conditions. Experimental models incorporating multiple concurrent risk factors may provide more clinically relevant insights.

## Conclusions

The available evidence suggests that high-fat diets and tobacco exposure adversely affect bladder health through overlapping metabolic and inflammatory mechanisms. High-fat intake promotes obesity-related oxidative stress, urothelial barrier disruption, and detrusor remodeling, leading to worsened lower urinary tract symptoms and increased bladder inflammation. Tobacco use, whether smoked or smokeless, directly injures the urothelium through carcinogens and reactive oxygen species, contributing to storage and voiding symptoms and substantially increasing bladder cancer risk. These exposures frequently coexist and may act synergistically to amplify bladder pathology. Overall, the available evidence suggests that the bladder may be a target organ of lifestyle-related injury; however, evidence for high-fat diet-related bladder effects in humans remains limited, and further human studies are needed. Integrating lifestyle interventions into clinical care and advancing research on metabolic-urothelial interactions may improve quality of life and reduce the burden of bladder dysfunction and malignancy.
